# Acetylsalicylic acid and salicylic acid present anticancer properties against melanoma by promoting nitric oxide-dependent endoplasmic reticulum stress and apoptosis

**DOI:** 10.1038/s41598-020-76824-6

**Published:** 2020-11-12

**Authors:** Priscila Ausina, Jessica R. Branco, Thainá M. Demaria, Amanda M. Esteves, João Gabriel B. Leandro, Alan C. Ochioni, Ana Paula M. Mendonça, Fernando L. Palhano, Marcus F. Oliveira, Wassim Abou-Kheir, Mauro Sola-Penna, Patricia Zancan

**Affiliations:** 1 Laboratório de Enzimologia e Controle do Metabolismo, Departamento de Biotecnologia Farmacêutica, Faculdade de Farmácia, Universidade Federal Do Rio de Janeiro, Rio de Janeiro, RJ 21941-902 Brazil; 2 Laboratório de Oncobiologia Molecular, Departamento de Biotecnologia Farmacêutica, Faculdade de Farmácia, Universidade Federal Do Rio de Janeiro, Rio de Janeiro, RJ 21941-902 Brazil; 3 Instituto de Bioquímica Médica Leopoldo de Meis, Universidade Federal Do Rio de Janeiro, Rio de Janeiro, RJ 21941-902 Brazil; 4 Department of Anatomy, Cell Biology and Physiological Sciences, Faculty of Medicine, American University of Beirut, Beirut, Lebanon

**Keywords:** Melanoma, Apoptosis, Autophagy, Stress signalling

## Abstract

Melanoma is the most aggressive and fatal type of skin cancer due to being highly proliferative. Acetylsalicylic acid (ASA; Aspirin) and salicylic acid (SA) are ancient drugs with multiple applications in medicine. Here, we showed that ASA and SA present anticancer effects against a murine model of implanted melanoma. These effects were also validated in 3D- and 2D-cultured melanoma B16F10 cells, where the drugs promoted pro-apoptotic effects. In both in vivo and in vitro models, SA and ASA triggered endoplasmic reticulum (ER) stress, which culminates with the upregulation of the pro-apoptotic transcription factor C/EBP homologous protein (CHOP). These effects are initiated by ASA/SA-triggered Akt/mTOR/AMPK-dependent activation of nitric oxide synthase 3 (eNOS), which increases nitric oxide and reactive oxygen species production inducing ER stress response. In the end, we propose that ASA and SA instigate anticancer effects by a novel mechanism, the activation of ER stress.

## Introduction

Endoplasmic reticulum (ER) stress is a physiological condition where the ER activates a series of reactions in response to protein accumulation, protein misfolding, or other stress signals, namely unfolded protein response (UPR), aiming to achieve intracellular protein homeostasis and, ultimately, survival^1,2^. This pathway is activated while cells are synthesizing proteins, particularly antibody-producing and proliferating cells^2^. Although, essentially a survival pathway, chronic or overwhelming acute ER stress can lead to cell death via apoptosis^1,2^. The UPR is composed of three independent signaling pathways, initiated by three ER transmembrane proteins, PRKR-like ER kinase (PERK), activating transcription factor 6 (ATF6), and inositol requiring protein 1α (IRE1α)^3,4^. These proteins, once activated, promote the synthesis of chaperones and lipids instigating degradation of proteins aiming to achieve protein homeostasis within the cell^3,4^. However, the three pathways also promote the expression of the transcription factor C/EBP homologous protein (CHOP), which signals cell growth arrest and apoptosis^3,4^.

Cancer cells are highly proliferative cells, so the metabolic engine is directed towards energy production in support of massive synthesis of nucleic acids, lipids, and proteins—the building blocks for cell division^5^. Among cancers, melanoma is one of the most aggressive, mainly due to its tendency to metastasize and therapy resistance^6^. Due to the overwhelming protein synthesis rate, ER stress and UPR are of great importance to the survival and maintenance of cancer cells^7,8^. Indeed, due to these characteristics, UPR is activated continuously in cancer cells, in a homeostatic balance to keep those cells viable under high stress^4,7^. However, it is known that interfering (either by inhibiting or activating) with ER stress in cancer cells, will strongly affect them physiologically and may lead to their death^3,4,7^. This was successfully reflected in pre-clinical approaches wherein interfering with ER stress presented a promising cancer therapy^7^, turning it into a target for controlling cancer at both the genetic and pharmacologic levels^9,10^. Accordingly, various drugs were produced to selectively inhibit the initial steps of UPR selectively, and results were promising whereby the selective therapy impeded the survival of cancer cells due to the inability to deal with such protein stress^9^. On the contrary, drugs that over activate UPR have been shown to induce apoptosis and have some beneficial anticancer effects^9^.

Salicylates are ancient drugs used for diverse medical purposes. They have multiple modes of action pertaining to their various therapeutic applications^11^. Although salicylic acid (SA) is the prototype form of the drug, the synthetic analog acetylsalicylic acid (ASA; aspirin) has been extensively used for more than a century for different conditions, from pain relief to blood pressure control^12^, due to its multiple targets. Although ASA is known and described to produce its analgesic effect by acetylating and inhibiting cyclooxygenase^12^, mechanisms of action relevant to many of its other beneficial effects are still poorly understood^11^. Over the years, extensive research has been conducted to decipher the underlying mechanisms of ASA, showing that it interferes with the expression of many proinflammatory modulators^13–15^, activates adenosine-monophosphate activated protein kinase (AMPK)^11^, inhibits phosphofructokinase^16^, among others. Additionally, salicylates, in general, are related to cell oxidative stress by both, generating reactive species as well as acting as a scavenger^17^. Some of these effects have been correlated to a putative anticancer effect of ASA and its metabolic product in humans^18,19^. Recently, these drugs have also been described to modulate ER stress in fibroblasts and adipocytes^20–24^.

The current work aims to study whether the deleterious effects of SA and ASA involve ER stress and to unveil the mechanisms by which it occurs. Moreover, this work aims to search for an anticancer effect of these drugs using an animal model for skin melanoma implants in mice.

## Results

**Figure 1:**
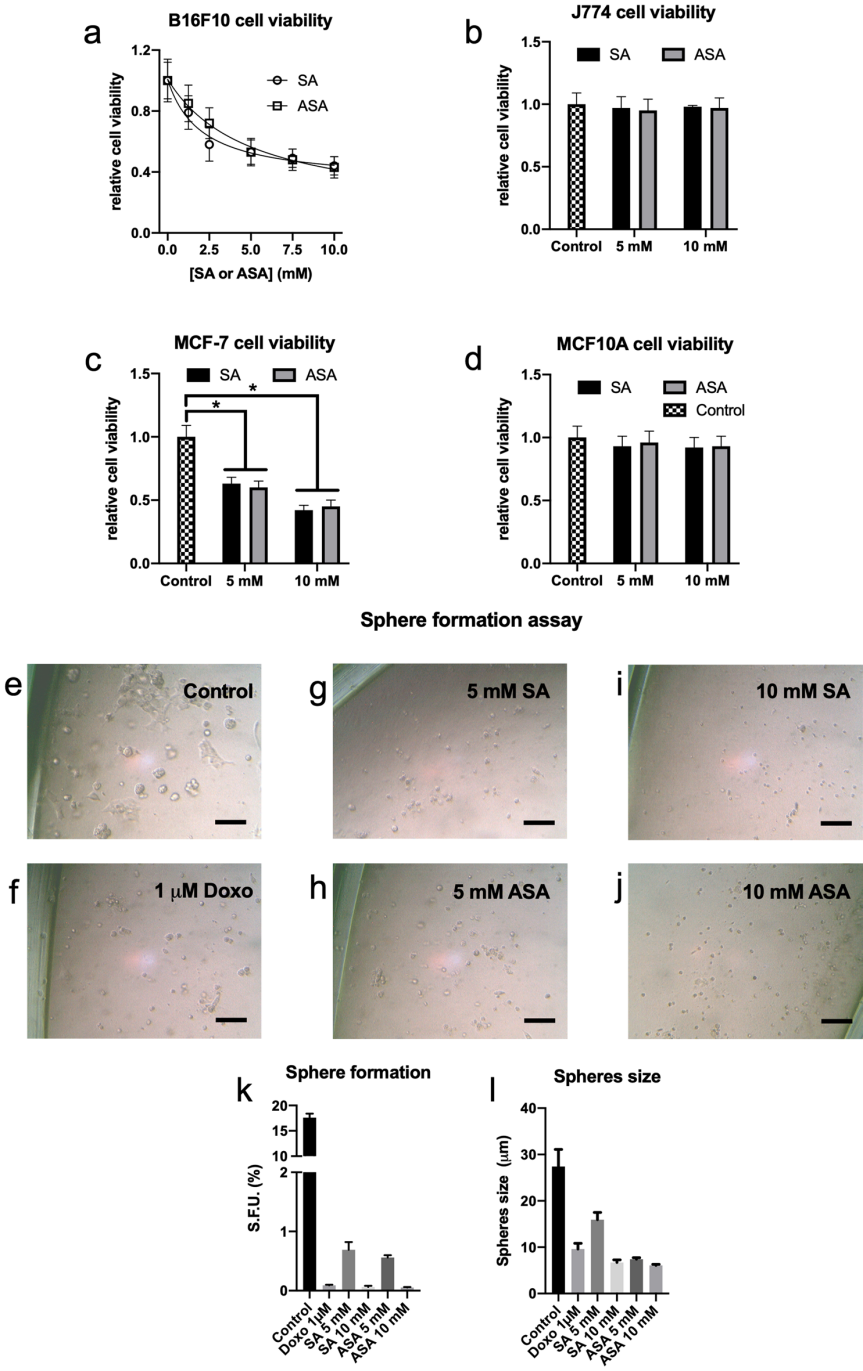
SA and ASA reduced cancer cells viability and impeded sphere formation capability in a 3D culture model. B16F10 (Panel **a**), J774 (Panel **b**), MCF-7 (Panel **c**) and MCF10A (Panel **d**) cells were grown in 2D cultures and treated with the concentrations of SA or ASA indicated on the abscissa for 24 h. These results are presented as the mean ± S.E.M of 4 independent experiments (n = 4). Panels **e**–**j**: Representative optical microscopy pictures of 3D-cultured B16F10 cells untreated (**b**) or treated with 1 µM doxorubicin (**c**), 5 mM SA (**d**), 5 mM ASA (**e**), 10 mM SA (**f**) and 10 mM ASA (**g**). Panels **k** and **l**: quantification of the numbers and the size, respectively, of spheres formed. These results are represented as mean ± S.E.M of 3 independent experiments (n = 3). * means P < 0.05 as compared to the control (One-way ANOVA followed by Dunnett post-test).

Initially, the anti-cancer effects of SA and ASA on B16F10 cells, a mouse-derived skin melanoma cell line, were evaluated in 2D and 3D cultured cells. Both, SA and ASA, promoted a dose-dependent decrease in 2D-cultured B16F10 cell viability, reaching an approximate 50% decrease at 5 mM of each drug (Fig. 1a). Increasing the concentration to 10 mM promoted a 55% decrease in cell viability, which was not statistically different from the effect of the drugs at 5 mM (Fig. 1a). As a control experiments, we tested the effects of these concentrations of the drugs on J774 murine non-tumor forming cell line. These cells were not responsive to either SA or ASA (Fig. 1b), indicating a selective effect of the drugs to the tumor cell line. This selectivity is confirmed since both drugs were efficient at reducing viability in a human breast cancer cell line, MCF-7 (Fig. 1c), but not in a non-tumor counterpart, MCF10A (Fig. 1d). The selective effects of SA and ASA on cancer cells has been demonstrated elsewhere and is compatible with the clinical use of these drugs to treat many diseases^25,26^. Moreover, these results show that the effects of the drugs are not specific to B16F10 melanoma cells but also affect other cancer cells lines, which has been shown elsewhere^27^.

The ability of cultured cancer cells to grow as nonadherent spheroids is a potent predictor of tumor growth and is currently used to evaluate potential anticancer agents^28–32^. Three-dimensional in vitro cell culture techniques enable modeling of tumors and their microenvironments to aid in cancer drug discovery^33–38^. So, we sought to use a Matrigel-based sphere formation assay protocol, that is previously designed by Bahmad et al.^34^, to assess the stem/progenitor cell-like properties of B16F10 cells upon exposure to either of the two treatments. B16F10 cells subjected to a sphere formation assay in nonadherent Matrigel-based conditions for 7 days yielded 17.8 ± 1% SFU (sphere forming units) under control conditions, with an average size of 27.4 ± 3.7 µm (Fig. 1e). Treatment with 5 or 10 mM SA or ASA resulted in a drastic reduction in the formation of SFU (Fig. 1g–j). In this case, there are significant differences between the effects of 5 and 10 mM of the drugs. In the presence of 5 mM SA (Fig. 1g), we observed a reduction in SFU reaching 0.6 ± 0.1%, contrasting with less than 0.1% in the presence of 10 mM SA (Fig. 1i and k). Similar results were obtained with ASA (Fig. 1h and j), where the effects did not differ from those obtained with SA (Fig. 1k). As a reference, B16F10 cells were treated with 1 µM doxorubicin (Fig. 1f) which reduced SFU to values similar to those obtained with 10 mM SA or ASA (Fig. 1k). The treatment with SA or ASA also significantly decreased the size of the spheres (Fig. 1l).

**Figure 2:**
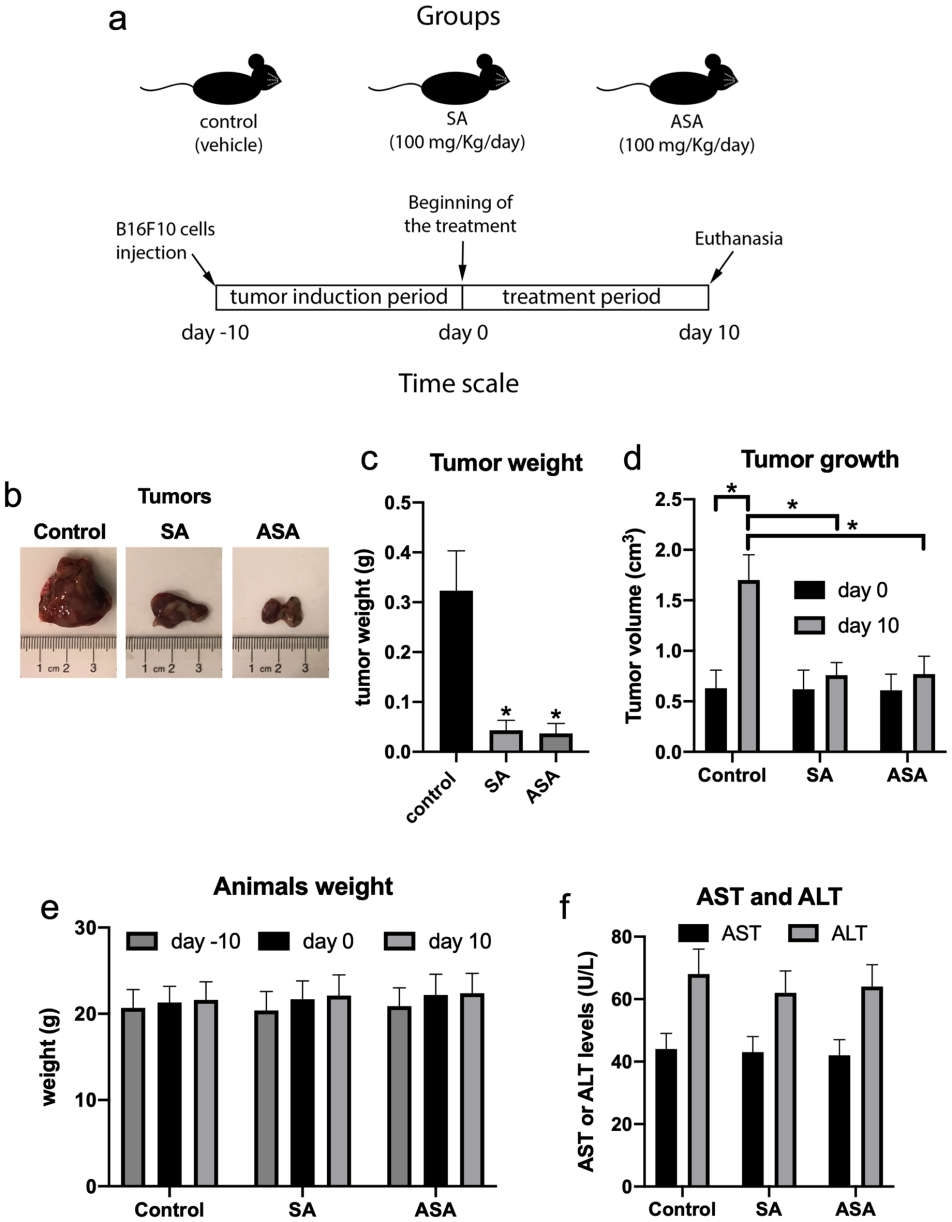
The anticancer effects of SA and ASA on a murine model of implanted melanoma. Panel **a**: design of the animal protocol. Panel **b**: representative pictures of the tumors extracted from untreated animals and those treated with SA or ASA. Panels **c** and **d**: tumors weight and tumors growth, respectively, comparing the control treatment with SA and ASA treatments. Panel **e**: average values for the mice body weight during the treatment. Panel **f**: average values for the activity of the liver enzymes AST and ALT in the serum of the animals. Values are mean ± S.E.M. of 8 different animals in each group (n = 8). * means P < 0.05 as compared to the control (One-way ANOVA followed by Dunnett post-test).

Next, we sought to evaluate the effects of SA and ASA drugs in vivo. B16F10 cells were subcutaneously implanted in the back of C57BL/6 J mice and allowed to form solid tumors for 10 days. After this period, mice were treated daily with PBS (vehicle) or 100 mg/kg SA or ASA by gavage for 10 consecutive days, after which animals were euthanized and tumors were removed, weighed, and processed for further analyses, as summarized in Fig. 2a. The extracted tumors were visually smaller in groups treated with SA or ASA, as compared to controls (Fig. 2b). Measuring the tumor weights after the sacrifice confirmed that the groups treated with SA or ASA were approximately 85% lighter, compared to control (Fig. 2c). The treatment indeed interfered with tumor growth, as evident by tumor volume at the beginning and the end of the treatment, wherein the tumors from animals treated with SA or ASA did not grow significantly (Fig. 2d). Additionally, SA and ASA did not interfere with mice development, as observed by the bodyweight of the animals (Fig. 2e), as well neither affected the serum levels of AST and ALT (Fig. 2f), indicating that the treatment did not injure the liver of the mice.

**Figure 3:**
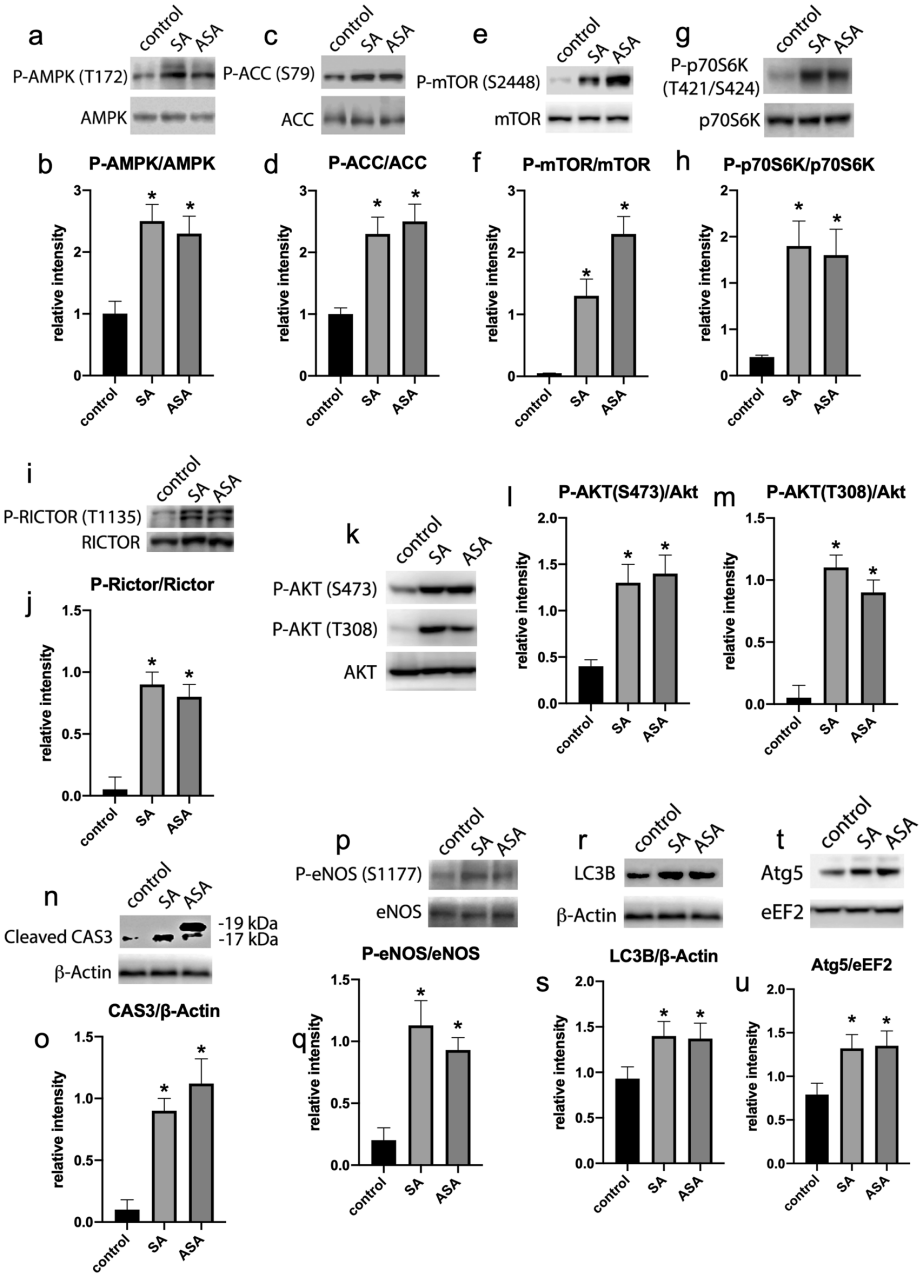
Signaling pathways profiles in the tumors extracted from animals treated or not with SA or ASA. Western blots displayed are the whole processed strips, as indicated in “Material and methods” and are representative samples of each group and represented in the graphics as mean ± S.E.M. of 8 different animals (n = 8). Panels **a** and **b**: AMPK and phospho-AMPK (T172). Panels **c** and **d**: ACC and phospho-ACC (S79). Panels **e** and **f**: mTOR and phospho-mTOR (S2448). Panels **g** and **h**: p70S6K and phospho-p70S6K (T421/S424). Panels **i** and **j**: Rictor and phospho-Rictor (T1135). Panels **k**, **l**, and **m**: Akt and phospho-Akt (T308 or S473). Panels **n** and **o**: cleaved CAS3 and ß-Actin (Different exposition periods for cleaved CAS3 are presented in supplementary material, Fig. S3). Panels **p** and **q**: eNOS and phospho-eNOS (S1177). Panels **r** and **s**: LC3B and ß-Actin. Panels **t** and **u**: Atg5 and eEF2. *Means P < 0.05 as compared to the control (One-way ANOVA followed by Dunnett post-test).

We were further interested in investigating the mechanism by which SA and ASA were interfering with tumor growth, we initially evaluated the activation of AMPK in the tumors, since it is described that SA, but not ASA, directly activates this enzyme^11^. AMPK activity was evaluated by measuring AMPK phosphorylation at T172, which is activating for the enzyme, and its substrate ACC, which is phosphorylated at S79 by AMPK. The tumors from animals treated with both, SA and ASA, showed increased phosphorylation of AMPK (Fig. 3a and b) and ACC (Fig. 3c and d), suggesting that both drugs are promoting AMPK activation in the tumors. These data tell us that the mechanism of AMPK activation within the tumors of treated animals is not due to the direct action of the drugs on the enzyme, since it has clearly been demonstrated that ASA does not present this property^11^. Curiously, we also found that tumors treated with both SA and ASA presented an increased phosphorylation of mTOR at S2448 (Fig. 3e and f), which is followed by an activation of mTORC1, which was evaluated by means of phosphorylation of its substrate, p70S6K at T421/S424 (Fig. 3g and h). Additionally, we found that the treatment with both drugs promoted the phosphorylation of Rictor at T1135 (Fig. 3i and j), which is a substrate for p70S6K^39^.

Since Rictor phosphorylation activates mTORC2, we evaluated phosphorylation of Akt at S473, which is classically phosphorylated by mTORC2^40^. Indeed, treatment with SA and SAS increased Akt phosphorylation at S473 confirming the activation of mTORC2 by the drugs (Fig. 3k and l). However, phosphorylation of Akt at T308 is also enhanced by the treatments with SA and ASA (Fig. 3k and m), indicating that the treatment might be also activating PDK1, as this is the enzyme phosphorylating Akt at T308. Indeed, it is demonstrated that Rictor activation, and thus mTORC2 activation, facilitates the phosphorylation of Akt at T308 by PDK1, explaining our results^41^. Recently, we have shown that simultaneous activation of AMPK, mTORC1, mTORC2 and Akt pathways in cancer cells activate cell apoptosis, leading to cancer cell death^42^. In our current study, we found that the treatment of the animals with SA and ASA promoted the cleavage of Caspase 3 (Fig. 3n and o), indicating that the drugs are triggering apoptosis and thus killing cancer cells. Although initially contradictory, activation of Akt (primarily a survival pathway) is known to kill cancer cells through the direct phosphorylation and activation of eNOS, leading to nitric oxide production and ultimately triggering apoptosis^43^. Here, we found that eNOS phosphorylation at S1177 was enhanced in the tumors from the animals treated with SA or ASA (Fig. 3p and q), suggesting that the above-described mechanism might be responsible for the effects those drugs are having on the tumors. Since AMPK activation is usually associated with the induction of autophagy, we evaluated this pathway through analyzing the levels of cleaved LC3B (Fig. 3r and s) and Atg5 (Fig. 3t and u) and found that autophagy was also being triggered by SA and ASA.

**Figure 4:**
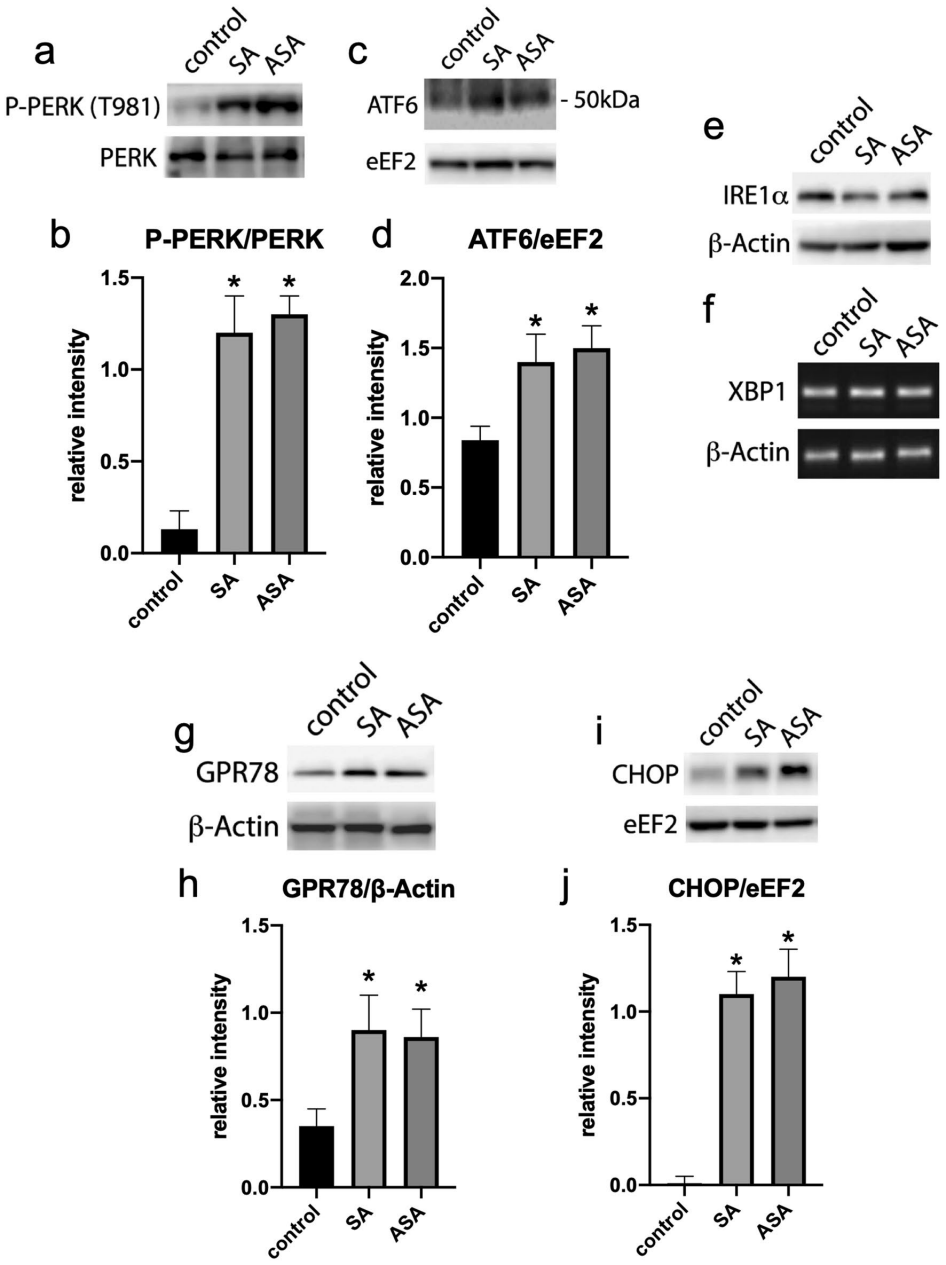
ER stress response evaluation in the tumors extracted from animals treated or not with SA or ASA. Western blots displayed are the whole processed strips, as indicated in “Material and methods” and are representative samples of each group and represented in the graphics as mean ± S.E.M. of 8 different animals (n = 8). Panels **a** and **b**: PERK and phospho-PERK (T981). Panels **c** and **d**: ATF6 and eEF2. Panel **e**: IRE1α. Panel **f**: XBP1. Panels **g** and **h**: GPR78 and ß-Actin. Panels **i** and **j**: CHOP and eEF2. *means P < 0.05 as compared to the control (One-way ANOVA followed by Dunnett post-test).

Activation of multiple pathways in cancer ultimately leading to apoptosis is being currently associated with the ER stress pathway, which also can be related to both, cell survival and cell death^44,45^. We then evaluated whether SA and ASA were triggering ER stress in the tumors of the treated animals. Treatment with both drugs increased the phosphorylation of PERK in the tumors of the animals (Fig. 4a and b), as well as promoted the cleavage of ATF6, assessed by evaluating the presence of the 50 kDa fragment of the protein in the tumors’ lysate (Fig. 4c and d). However, we did not detect differences in IRE1α phosphorylation, since no Western Blot migration shift was detected for this protein (Fig. 4e); besides, we did not detect the splicing of its downstream effector, XBP1 (Fig. 4f). On the other hand, the ATF6 downstream effector GPR78 was upregulated in the tumors of treated animals (Fig. 4g and h), as well as the PERK downstream effector CHOP (Fig. 4i and j). These results suggest that the drugs are triggering ER stress response through PERK and ATF6 pathways.

To better understand the cellular mechanisms involved in SA and ASA anticancer effects, we tested the effects of the drugs directly on B16F10 cell cultures. As we have described above, SA and ASA decreased B16F10 viability in a dose-dependent manner, reaching 45% viability with 10 mM (Fig. 1a). These drugs impacted B16F10 cell proliferation similarly, where the treatment of the cells with 10 mM SA or ASA for 24 h decreased the rate of cell proliferation by approximately 50% (Fig. S1a), and increased cell permeability to DNA dye 7-AAD indicating increased plasma membrane permeability (Fig. S1b). Additionally, treatment of the cells with 10 mM SA or ASA for 24 h increased the labeling of these cells with anti-Annexin V antibody (Fig. S1c), indicating that cells are undergoing apoptosis and support the findings presented in Fig. 3n and o for caspase 3 cleavage for the in vivo model. Autophagy was also triggered in B16F10 cells treated for 24 h in the presence of 10 mM SA or ASA (Fig. S1d), confirming the LC3 cleavage shown for the tumors of the animals treated with the drugs (Fig. 3r and s). This might be a consequence of AMPK activation, which we also observed in B16F10 cells treated with SA or ASA by means of the increased phosphorylation of the enzyme at T172 (Fig. S1e and f.) and confirmed by the increased phosphorylation of ACC at S79 (Fig. S1g and h). This activation was similar to those observed in the tumors of the animals treated with these drugs (Fig. 3a–d).

B16F10 cells treated with 10 mM SA or ASA also exhibited boosting of mTORC1 and mTORC2 activity. This conclusion was reached after a series of experiments. First, the drugs promoted the phosphorylation of mTOR at S2448 (Fig. S1i and j), which is indicative of both complexes’ activation. However, we also observed the increased phosphorylation of two of mTORC1 substrates, p70S6K at T421/S424 (Fig. S1k and l) and Rictor at T1135 (Fig. S1m and n). Rictor is linked to mTORC2, and once phosphorylated, it activates this complex. This is confirmed by the increased phosphorylation of Akt at S473 (Fig. S1o and p). This enzyme is also hyperphosphorylated at T308 (Fig. S1o and q), which might be the mechanism of mTORC1 activation by these drugs. Results obtained with the B16F10 cell line treated with 10 mM SA or ASA confirm the results we showed with the tumors from treated animals (Fig. 3). Moreover, Akt also phosphorylates and activates eNOS, which we showed with the tumors (Fig. 3p and q) and confirmed here (Fig. S1r and s). To confirm that SA and ASA are activating eNOS, we measured NO production by B16F10 cells treated with 10 mM SA or ASA, and we observed a sevenfold increase in the levels of NO (Fig. S1t). This is indicative that the drugs are promoting oxidative stress, which is confirmed by DCFDA staining of the cells (Fig. S1u), and might be linked to cell death and the ER stress. However, NO is not the only reactive species produced upon the treatment of the cells with the drugs. We also observed an increase in ROS, as shown in Fig. S1v.

Since oxidative stress might also be linked to ER stress, which we observed in the tumors treated with SA and ASA, we evaluated whether the drugs trigger ER stress in cultured B16F10 cells. Indeed, we found similar results to those presented in Fig. 4. Both drugs promoted the phosphorylation of PERK on T981 (Fig. S2a and b), as well as increased expression of ATF6 (Fig. S2c and d). However, no effect was observed on IRE1α phosphorylation (Fig. S2e) nor its downstream readout, the splicing of XBP1 (Fig. S2f.; 1 µM thapsigargin – TG – was used as a positive control for XBP1 splicing). Conversely, we confirmed that the drugs activate PERK and ATF6 pathways by evaluating their own downstream effector's CHOP (Fig. S2g, h, and i) and GPR78 (Fig. S2j and k). Additionally, we also confirmed that ATF4, a downstream effector of the PERK pathway directly involved in the transcription of CHOP, was upregulated by both drugs in cultured B16F10 cells (Fig. S2l).

**Figure 5:**
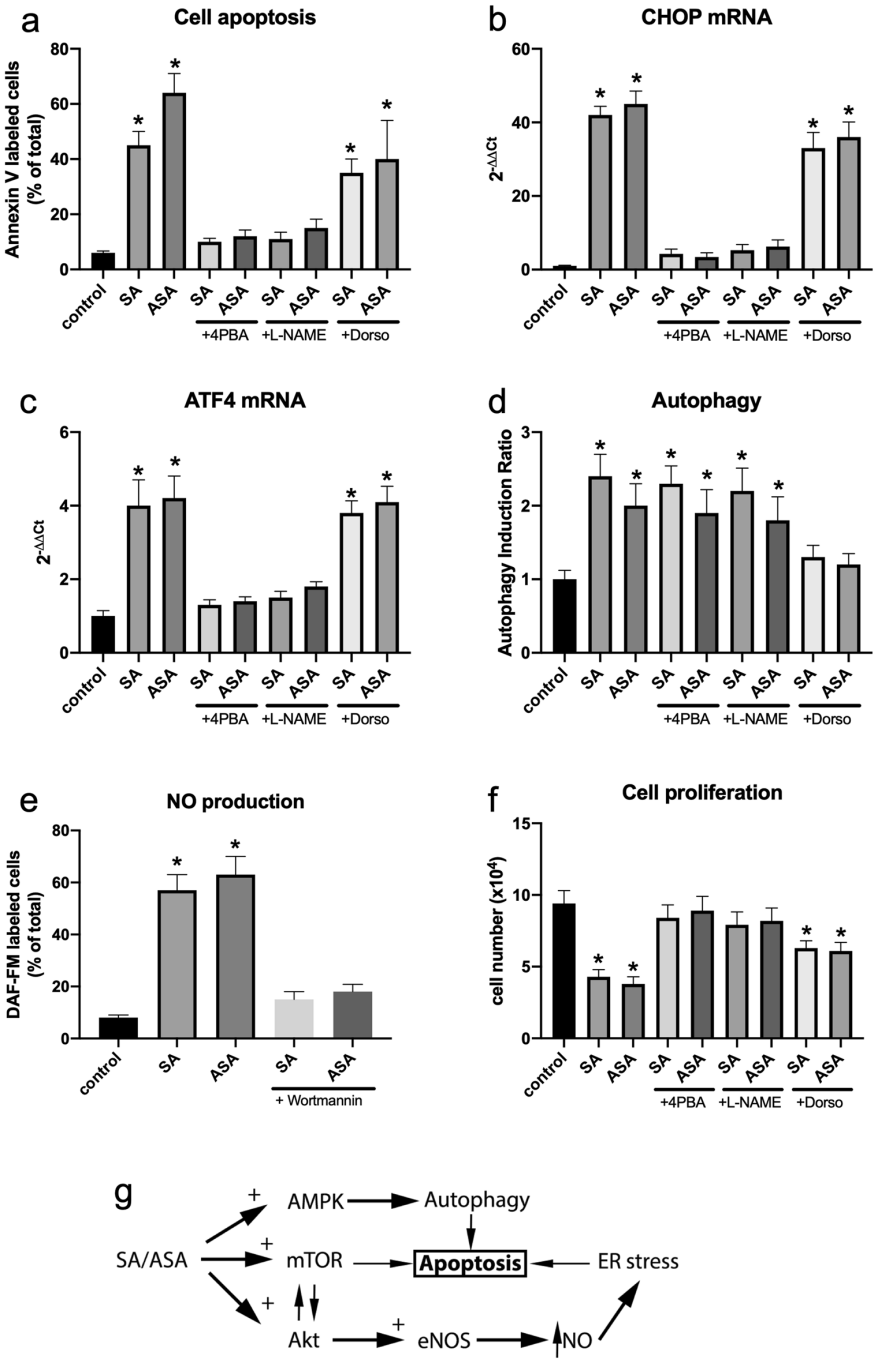
Reversal of SA and ASA effects on B16F10 cells by the inhibitor of the signaling pathways. Plotted values are mean ± S.E.M. of 3–4 independent experiments (n = 3–4). Panel **a**: apoptosis. Panel **b**: CHOP mRNA levels. Panel **c**: ATF4 mRNA levels. Panel **d**: autophagy. Panel **e**: NO production. Panel **f**: cell proliferation. Panel **g**: scheme for SA and ASA action. *means P < 0.05 as compared to the control (One-way ANOVA followed by Dunnett post-test).

GPR78 and CHOP are directly involved in triggering apoptosis in diverse cellular systems. Thus, we tested whether their upregulation is involved in the SA/ASA-induced apoptosis of B16F10 cells. Indeed, treatment of B16F10 cells with 4-PBA, which blocks ER stress response, prevented B16F10 cells to undergo apoptosis upon the treatment with SA or ASA (Fig. 5a). Since it is described that NO might trigger ER stress, we evaluated whether L-NAME, an inhibitor of eNOS, would diminish the effects of SA and ASA on B16F10 cells. Our results showed that L-NAME also prevents SA/ASA-induced apoptosis in B16F10 cells (Fig. 5a). As evaluated by qPCR, L-NAME also prevented the upregulation of CHOP and ATF4 (Fig. 5b and c, respectively), indicating that NO is triggering SA/ASA-induced ER stress. On the other hand, dorsomorphin, an inhibitor of AMPK, prevented SA/ASA-induced autophagy (Fig. 5d), but not the upregulation of the ER stress markers, CHOP, and GPR74 (Fig. 5b and c, respectively), nor apoptosis (Fig. 5a). By inhibiting Akt activation with Wortmannin, we prevented SA/ASA-induced NO production (Fig. 5e), showing that Akt is responsible for eNOS phosphorylation and activation. In the end, we assessed cell proliferation, and we observed that the inhibition of cell proliferation induced by both SA and ASA, is prevented by L-NAME and partially prevented by dorsomorphin (Fig. 5f). Therefore, we concluded that Akt and mTOR are being activated by SA and ASA, leading to the activation of eNOS which promotes NO-triggered ER stress. Simultaneously, the drugs are promoting AMPK-triggered autophagy ultimately leading to cell apoptosis (Fig. 5g).

## Discussion

In the current work, we showed that SA and ASA present anticancer effects against skin melanoma implanted on C57BL6/J mice. The anticancer effects of ASA have been previously discussed in the literature, but its actual therapeutic use is still controversial^18,46,47^. More is discussed about a putative chemopreventive action of ASA, such as its use to prevent myocardial infarct, and a reduced incidence of metastasis in cancer patients regularly taking the drug, than a direct effect reducing existent tumors^18,46–48^. The results that we presented here showed that the treatment of mice with ASA, or even its metabolic product, SA, also prevented implanted cancer growth, which is beneficial especially when associated with other therapies. The cancer model that was used here was produced using the B16F10 cell line. This particular cell line, derived from mice melanoma, is highly proliferative and, once implanted in mice, generates aggressive tumors that rapidly metastasize^49,50^. For these reasons, we used a relatively short-term protocol of cell implantation and tumor growth (10 days). At the conclusion of the protocol, there was no evidence of metastasis, in the lymph nodes lungs, liver, or intestines. Using a similar approach, in an 18–22 days protocol, Harrell et al. also did not observe metastasis but only increased lymph flux and activity^49^. We are aware that the use of other melanoma cell line, such as YUMM cell lines^51^, would greatly improve the confidence of SA/ASA effects on melanoma, but such cells lines were not available for a series of issues including the current restraints imposed by COVID-19 pandemic. Therefore, this should be considered a limitation of the present study.

The mechanism by which SA and ASA present their anticancer effects involves multiple pathways, and not all of them have been scrutinized in the current work. Our observations reveal that there is an activation of the Akt/mTOR pathway, which awkwardly coincides with AMPK activation. This latter has been elegantly demonstrated to be directly due to the binding of salicylate at an AMPK activation site, which promotes allosteric activation and inhibition of the dephosphorylation of the enzyme at S172^11^. Although it has been shown that AMPK activation is achieved by salicylate and not by acetylsalicylate^11^, it is already known that tumor cells, such as the liver, rapidly metabolize acetylsalicylate to form salicylate^14^, the reason why many published works on cancer test both drugs.

In the current work, we observed that in both in vivo and in vitro models, AMPK is phosphorylated and activated upon treatment with SA or ASA. However, by preventing SA/ASA-induced AMPK activation using dorsomorphin, there was only a partial reversal of the effects of the drugs on B16F10 cells viability. This suggests that AMPK activation is only partially involved in the anticancer effects of SA and ASA. Curiously, AMPK activation is usually related to cell survival pathways where AMPK plays a role in protecting cancer cells from metabolic oxidative stress^52^. On the other hand, activation of AMPK can also negatively interfere with cancer cells either by arresting cell growth or by priming cancer cells to be more sensitive to death signals^53^. This latter effect is usually linked to an AMPK-dependent inhibition of mTOR^53,54^, which is involved in another survival pathway within many cells^54^. However, in the current work, we found that the mTOR pathway is also activated upon treatment with SA or ASA. This activation involves both mTOR complexes, mTORC1 and mTORC2, as evaluated by means of the phosphorylation of specific substrates of each complex. Additionally, we found that the upstream effector of mTORC1 and downstream effector of mTORC2, Akt^55^, is also activated upon SA and ASA treatment, which is in accordance to some recently published results^56^. Usually, Akt activation is also related to cell survival due to its antiapoptotic effects^57^. However, in the current work, we have strong evidence supporting the theory that apoptosis is triggered and involved in the anticancer effects of SA and ASA even though the drugs are promoting Akt activation.

Our results suggest activated Akt, mTOR and AMPK might be associated with cellular oxidative stress via activation of the nitric oxide generation enzyme eNOS^58–60^. These effects are also linked to the generation of ROS^59^, ER stress and UPR^58^, and induction of cell apoptosis^61^. Indeed, what we saw here is that, upon the treatment of either tumors induced in animals or B16F10 cells with SA or ASA, eNOS was activated (assessed by means of the phosphorylation of the enzyme at S1177) and ER stress and UPR were activated through PERK and ATF6 branches. Additionally, the final modulator of these pathways, the pro-apoptotic transcription factor CHOP, was upregulated following treatment with the drugs. The ER stress-mediated upregulation of CHOP has been associated with the induction of apoptosis in many cellular systems and under different signaling conditions, which confers a pro-apoptotic effect to this transcription factor^1,62^. Usually, this final effect occurs when ER-stress is stably activated or is unable to resolve the oxidative stress due to misfolded protein accumulation^1,62^. Here, studying the mechanism of SA/ASA action in B16F10 cells, we observed increased oxidative stress, particularly by augmented NO and ROS production. Therefore, this increased oxidative stress might be contributing to the final effect on the tumors that is the arrest of growth and induction of death. Indeed, we found that NO is directly involved in the process, since inhibiting eNOS in SA/ASA-treated B16F10 cells prevented the upregulation of CHOP and ATF4, and therefore preventing apoptosis trigger. The anticancer effects of ASA on melanoma have been discussed elsewhere^63–68^, but it is the first time that involvement of UPR is presented as a mechanism for this drug anticancer property.

Although most of the papers dealing with implanted tumor cells in mice use immune-compromised animals, here we opted to use wild-type immune-competent C57BL6/J mice since B16F10 cells are from the same animal origin and would not be rejected by the mice. We believe that this animal model is more robust to evaluate anticancer drugs due to the key role of immune system on cancer development and progression. On the other hand, since SA/ASA have modulatory effects on inflammation and, therefore, on immune response, it is possible that part of the effects observed here are not only due to the drugs action on cancer cells but also on immune system. For instance, using an immune-compromised mouse model, Hammerlindl et al. did not observe anticancer effects of ASA alone (100 mg/Kg, such as the current work) against different implanted human melanoma cells^69^. However, these authors have observed that ASA strongly enhanced cytotoxicity of otherwise ineffective sorafenib dosages^69^. Sorafenib, which is a tyrosine kinase inhibitor^70^, also induces autophagy^71^, which is involved on its anticancer effects^72^. Sorafenib also mimics some of the immune-mediated response even in immune-deficient mice (NOD/SCID) improving the control of hepatocarcinoma^73^. Therefore, we can suggest that in the Hammerlindl et al. work, ASA did not act alone due to the lack of immune response, which was partially mimicked by the combination of sorafenib. Although it is only a guess, this hypothesis is supported by other works showing anticancer effects of ASA alone, using immune competent C57BL6/J mice^27,74^. Moreover, in spite of the importance of immune system to SA/ASA anticancer effects, it is clear by the current work that these drugs present cellular effects on cancer cells that are promoting these cells death (in vitro) and controlling cancer growth (in vivo). It is also true that the use of immune-compromised animals, in addition to implantation of human melanoma cells, would have confirmed the importance of the direct cancer cell effects of ASA to its anticancer properties and improved the current work and, thus, should be considered a second limitation of this work.

In conclusion, our results suggest that SA and ASA might present promising anticancer effects on melanoma cells by triggering ER stress-induced apoptosis through upregulation of NO production via Akt/mTOR/AMPK-activated eNOS action.

## Materials and methods

All the experimental animal protocols were previously approved by the Animal Care and Use Committee from the Health Sciences Center of the Federal University of Rio de Janeiro (CEUA/CCS/UFRJ 109/15).

All methods described in the present work were carried out in accordance with relevant and up-to-date guidelines and regulations.

### Cells

All cells source and protocols was such as described previously by Sola-Penna et al^75^. The mouse-derived skin melanoma cell line, B16F10, and mouse monocyte macrophage cell line, J774, and human breast cancer cell line, MCF-7, were obtained from the Cell Bank of Rio de Janeiro (www.bcrj.org.br Duque de Caxias, RJ, Brazil) and were grown and maintained in Dulbecco's Modified Eagle’s Medium (DMEM) with 25 mM glucose supplemented with 10% (vol/vol) heat-inactivated Fetal Bovine Serum (FBS) and 5 mM L-glutamine (Invitrogen, São Paulo, SP, Brazil) at 37 °C and 5% CO_2_ humidified incubator. The MCF10A lineage, a non-tumorigenic human breast cell line kindly gifted by Prof. Mitzi Brentani (Universidade de São Paulo, São Paulo, Brazil), was grown and maintained at 37 °C (5% CO_2_ atmosphere) in DMEM/F12 medium with 25 mM glucose supplemented with 10% (vol/vol) FBS, 0.02 mg/ml EGF, 5 mg/ml insulin, 1.25 mg/ml hydrocortisone, 0.1 mg/ml cholera toxin, and 5 mM L-glutamine (Invitrogen, São Paulo, SP, Brazil)^75^.

### Animals and tumor-inducing and treatment

The animal protocol was performed accordingly to what was previously approved by the Animal Care and Use Committee from the Health Sciences Center of the Federal University of Rio de Janeiro (CEUA/CCS/UFRJ 109/15). Male C57BL6/J mice of 6 weeks old, were individually housed at the animal facilities of the Pharmacy School/UFRJ, under 12 h/12 h light/dark cycle, when they had ad libitum access to chow and water.

A group of 24 animals was injected in the back with 2 × 10^5^ B16F10 cells suspended in 50 µL PBS, which formed a solid tumor with approximately 0.6 ± 0.2 cm^3^ after 10 days. Then, mice were randomly divided into three groups and daily treated with PBS or 100 mg/kg SA or ASA (using 30 mg/mL buffered solution), always by gavage at the beginning of the light cycle. After 10 days of treatment, the tumors were measured with a caliper and mice were sacrificed. Most of their organs, blood, plasma, serum, and the tumors were immediately weighed and frozen in liquid N_2_. Before being analyzed, tumors were crunched in liquid N_2,_ and the powdered material was stored at -80 °C and used for Western blot and qPCR analyses. Serum blood samples were used to evaluate glucose, aspartate transaminase (AST), and alanine transaminase (ALT) using commercial kits for these purposes (Doles Reagentes, Panamá, GO, Brazil). The volumes of the tumors during the protocol were calculated based on the caliper measurements, according to the described in the literature^76^.

### Cell viability

To assess B16F10 cells viability, 8,000 cells were seeded in 96-well plates, incubated at 37 °C and 5% CO_2_ for 24 h, and allowed to reach 70% confluency before the treatments begin. Cells were treated with different concentrations of SA or ASA for 24 h and then the media were removed and cell viability was evaluated by the 3-(4,5-dimethylthiazol-2-yl)-2,5-diphenyltetrazolium bromide (MTT) assay according to the manufacturer’s protocols^77^. Cell proliferation was calculated using the percentage of the optical density (OD) ratio of treated cells relative to control (media without treatment). Data represent the average of three experiments.

### Sphere formation assay

Sphere formation assay was done as described by Bahmad et al., 2018^34^. In brief, single-cell suspensions were mixed in a 50 μL volume of 1:1 cold growth factor-reduced Matrigel (BD Biosciences)/growth medium, in duplicates, at a density of 2,000 cells/well. We plated this cell suspension around the rim of each well of a 24‑well plate and left them for 60 min to solidify at 37 °C in a 5% CO_2_ humidified incubator. Then, 500 μL of DMEM cell growth medium, with or without treatment, was gently added to the center of each well and replenished every 2–3 days. At 7 days after plating, formed spheres are counted and assessed using the sphere formation efficiency or sphere formation unit (SFU) formula: SFU (in %) = (number of spheres counted ÷ number of input cells) × 100. Average diameter of spheres was also evaluated for the different conditions (average of 30 spheres per condition from three independent experiments), and Bel Inv100 microscope (Bel Engineering, Monza, Italy) was used for the acquisition of bright field images of the cultured spheres and BELView software (Bel Engineering, Monza, Italy) was used to analyze the results.

### Western blotting

For Western blot, all the samples were prepared in mild-RIPA buffer^78^ supplemented with protease inhibitor cocktail (Sigma-Aldrich, St. Louis, MO, USA). For the tumors’ samples, approximately 30 mg of tumor powders were directly mixed with 250 µL of the aforementioned buffer. After vigorous vortex, the mixture was centrifuged (10 min, 800 x*g*) to remove debris and protein content was evaluated using a commercial kit (Pierce BCA Protein Assay Kit, ThermoFischer, Carlsbad, CA, USA). For B16F10 cells, the cells were seeded in 6-well plates (10^5^ cells/well) and grown to approximately 70% confluency, after which the media were removed and cells were treated according to the experiments. After the treatments, the media were removed and cell proteins were extracted with the above mentioned mild-RIPA buffer, following the same procedures described for the tumors’ samples. Protein extracts were diluted into submitted to SDS-PAGE loading buffer and submitted to electrophoresis^79^, followed by overnight transfer to nitrocellulose membranes at 30 V. Membranes were stained with Ponceau S, processed by cutting the appropriate regions for the specific proteins and conditions and the resulting membranes strips were de-stained by washing with distilled water. Then, the membranes strips were incubated overnight with the following antibodies: anti-β-actin (dilution 1:1000, Cat# 4967, Cell Signaling Technology, Danvers, MA, USA), anti-ACC (dilution 1:1000, Cat# 3662, Cell Signaling Technology, Danvers, MA, USA), anti-phospho-ACC (S79) (dilution 1:1000, Cat# 3661, Cell Signaling Technology, Danvers, MA, USA), anti-Akt (dilution 1:1000, Cat# 9272, Cell Signaling Technology, Danvers, MA, USA), anti-phospho-Akt (T308) (dilution 1:1000, Cat# 9275, Cell Signaling Technology, Danvers, MA, USA), anti-AMPKα (dilution 1:1000, Cat# 2532, Cell Signaling Technology, Danvers, MA, USA), anti-phospho-AMPKα (T152) (dilution 1:1000, Cat# 2535, Cell Signaling Technology, Danvers, MA, USA), anti-ATF6 (dilution 1:1000, Cat# sc-22799, Santa Cruz Biotechnology, Santa Cruz, CA, USA), anti-CHOP (dilution 1:1000, Cat# sc-575, Santa Cruz Biotechnology, Santa Cruz, CA, USA), anti-Cleaved Caspase 3 (CAS3) (D175) (dilution 1:1000, Cat# 9661, Cell Signaling Technology, Danvers, MA, USA), anti-eEF2 (dilution 1:1000, Cat# 2332, Cell Signaling Technology, Danvers, MA, USA), anti-eNOS (dilution 1:1000, Cat# 8331, Cell Signaling Technology, Danvers, MA, USA), anti-phospho-eNOS (S1177) (dilution 1:1000, Cat# sc-12972, Santa Cruz Biotechnology, Santa Cruz, CA, USA), anti-GPR78 (dilution 1:1000, Cat# sc-13968, Santa Cruz Biotechnology, Santa Cruz, CA, USA), anti-IRE1α (dilution 1:1000, Cat# sc-20790, Santa Cruz Biotechnology, Santa Cruz, CA, USA), anti-LC3B (dilution 1:1000, Cat# 3868, Cell Signaling Technology, Danvers, MA, USA), anti-mTOR (dilution 1:1000, Cat# 2972, Cell Signaling Technology, Danvers, MA, USA), anti-phospho-mTOR (S2448) (dilution 1:1000, Cat# 2971, Cell Signaling Technology, Danvers, MA, USA), anti-p70S6K (dilution 1:1000, Cat# 9202, Cell Signaling Technology, Danvers, MA, USA), anti-phospho-p70S6K (T421/S424) (dilution 1:1000, Cat# 9204, Cell Signaling Technology, Danvers, MA, USA), anti-PERK (dilution 1:1000, Cat# sc-13073, Santa Cruz Biotechnology, Santa Cruz, CA, USA), anti-phospho-PERK (T981) (dilution 1:1000, Cat# sc-32577, Santa Cruz Biotechnology, Santa Cruz, CA, USA), anti-Rictor (dilution 1:1000, Cat# 2114, Cell Signaling Technology, Danvers, MA, USA), anti-phospho-Rictor (T1135) (dilution 1:1000, Cat# 3806, Cell Signaling Technology, Danvers, MA, USA). After incubation with the primary antibodies, membranes strips were washed and treated for 1 h with the following secondary antibody accordingly to the source of primary antibody: peroxidase-affinipure goat anti-mouse IgG (dilution 1:10,000, Cat# 115–035-146, Jackson ImmunoResearch Labs, West Grove, PA, USA) and peroxidase-affinipure goat anti-rabbit IgG (dilution 1:10,000, Cat# 115–035-144, Jackson ImmunoResearch Labs, West Grove, PA, USA). After this incubation, membranes were washed and developed using Amersham ECL Western Blotting Reagent (Cat# RPN2124, GE Healthcare Bio-Sciences, Pittsburg, PA, USA). Staining was evaluated using C-DiGit Blot Scanner (LiCor, Lincoln, NE, USA), and quantifications of the blots were performed using the software Image J64 (https://imagej.nih.gov/ij NIH, USA). All the regions shown in the current paper are the result of complete exposition of the cut membrane strips.

### RT-PCR and RT-qPCR

Total RNA was extracted from tumor samples or B16F10 cells following the same procedure described for protein extraction, but using 500 µl of Trizol reagent (ThermoFischer, Carlsbad, CA, USA) to extract RNA, following the manufacturers’ indication. Total RNA was quantified using a Picodrop Pico100 apparatus (Picodrop Limited, Hinxton, UK). cDNA synthesis was performed using the High-capacity cDNA Reverse Transcription Kit (ThermoFischer, Carlsbad, CA, USA). RNA and cDNA qualities were evaluated by running agarose gels according to the previously described protocols^80^. For RT-PCR, 100 ng cDNA were submitted to 30 cycles PCR using the AccessQuick RT-PCR System kit (Cat# A1703, Promega, Fitchburg, WI, USA), following the manufacturer directions. The primers used for RT-PCR were: XBP1 forward primer: 5ʹ-ACACGCTTGGGAATGGACAC-3ʹ, reverse primer: 5ʹ-CCATGGGAAGATGTTCTGGG-3ʹ; ß-actin (control gene): forward primer: 5ʹ-CTCAGGAGGAGCAATGATCTTGAT-3ʹ, reverse primer: 5ʹ-TACCACCATGTACCCAGGCA-3ʹ^81^. The program for all amplifications was 2 min at 94 °C followed by 30 cycles of 30 s at 94 °C, 30 s at 60 °C and 30 s at 72 °C, then additional 10 min at 72 °C. The products of RT-PCR amplification were then submitted to agarose gel electrophoresis (3%) containing the UniSafe Dye (Uniscience Corp. Miami Lakes, FL, USA). For RT-qPCR, 10 ng of the cDNA were submitted to 40 cycles PCR using the GoTaq qPCR Master Mix (Cat# A6002, Promega, Fitchburg, WI, USA) following the manufacturer directions, and amplification was detected in a QuantStudio 5 (ThermoFischer, Carlsbad, CA, USA). The program for all amplifications was 2 min at 95 °C followed by 40 cycles of 15 s at 95 °C and 1 min at 60 °C. A dissociation curve was performed at the end of the experiment and dissociation peak was analyzed. The fold expression was calculated by the 2^−ΔΔCt^ method, as described previously^82^. The primers used were: AFT4: forward primer: 5ʹ-ACATTCTTGCAGCCTTTCCC-3ʹ, reverse primer: 5ʹ-TAAGCAGCAGAGTCAGGCTT-3ʹ, 128 bp, 97% efficiency^83^; CHOP: forward primer: 5ʹ-CTGCCTTTCACCTTGGAGAC-3ʹ, reverse primer: 5ʹ-CGTTTCCTGGGGATGAGATA -3ʹ, 118 bp, 103% efficiency^84^; HPRT1 (reference gene): forward primer: 5ʹ-CCCTGGTTAAGCAGTACAGCCCC-3ʹ, reverse primer: 5ʹ-AGTCTGGCCTGTATCCAACACTTCG-3ʹ, 90 bp, 97% efficiency^80^.

### Cell cytometry

The assays for cell proliferation, 7-Aminoactinomycin D (7-AAD) staining, apoptosis, autophagy, oxidative stress, reactive oxygen species (ROS) production, and nitric oxide production were evaluated by cell cytometry using the Muse apparatus (Muse Cell Analyzer, Merck Millipore, Billerica, MA, USA). For these experiments, cells were seeded in 24-well plates (4 × 10^4^ cells/well) and then incubated for 24 h until reaching almost 70% confluency. Then, cells were treated accordingly and labeled with different dyes depending on the experiment. Cell proliferation was evaluated as the average of the differences in the number of total cells between the beginning and the end of all the experiments and is a representation of three different experiments. 7-ADD and PE Annexin V reagents were from BD Pharmingen (BD Biosciences, Franklin Lakes, NJ, USA). Autophagy was evaluated using the Autophagy kit from Muse (Muse Cell Analyzer, Merck Millipore, Billerica, MA, USA). The oxidative stress detection dye, 2′,7′-dichlorodihydrofluorescein diacetate (DCFDA) and the nitric oxide (NO) detection reagent, 4-Amino-5-Methylamino-2′,7′-Difluorofluorescein Diacetate (DAF-FM) were purchased from ThermoFischer (ThermoFischer, Carlsbad, CA, USA), and the ROS detection dye, Dihydroethidium (DHE) were from Cayman Chemical (Ann Arbor, MI, USA). For DCFDA labeling, cells were incubated in the presence of 50 µM DCFDA for 30 min before the experiments. DAF-FM (1 µM) and DHE (1 µM) were added to the suspended cells 15 min before readings. All the protocols followed were according to the manufacturers’ directions.

### Data analyses and statistics

All graphics and statistical analyses were performed with software Prism 8 for Mac (GraphPad Software Inc, La Jolla, CA, USA). The obtained data followed a Normal distribution, as evaluated. One-way ANOVA followed by Dunnett’s post-test, was used to determine the significance of the differences. Statistical significance was reported when the P-value was less than 0.05 (*P < 0.05).

### Ethics approval

The animal protocol used for the current work was performed accordingly to what was previously approved by the Animal Care and Use Committee from the Health Sciences Center of the Federal University of Rio de Janeiro (CEUA/CCS/UFRJ 109/15).

## Supplementary information

Supplementary Information 1.

## Data Availability

The raw data from the current work are available for academic purpose upon request to the corresponding author.
