# Supplementary material for: Acetylsalicylic acid and salicylic acid present anticancer properties against melanoma by promoting nitric oxide-dependent endoplasmic reticulum stress and apoptosis
Source: Sci Rep. 2020 Nov 12;10:19617. doi: 10.1038/s41598-020-76824-6 (PMC7665072; doi:10.1038/s41598-020-76824-6)
Supplement: Supplementary file 1 — Supplementary Information 1. [file 41598_2020_76824_MOESM1_ESM.pdf]

Supplementary material

Acetylsalicylic acid and salicylic acid present anticancer properties against melanoma by promoting nitric oxide-dependent endoplasmic reticulum stress and apoptosis

Priscila Ausina[1], Jessica R. Branco[2], Thainá M. Demaria[1], Amanda M. Esteves[2], João Gabriel B. Leandro[1], Alan C. Ochioni[2], Ana Paula M. Mendonça[3], Fernando L. Palhano[3], Marcus F. Oliveira[3], Wassim Abou-Kheir[4], Mauro Sola-Penna[1], Patricia Zancan[2,*]

[1]Laboratório de Enzimologia e Controle do Metabolismo, Departamento de Biotecnologia Farmacêutica, Faculdade de Farmácia, Universidade Federal do Rio de Janeiro, 21941-902, Rio de Janeiro, RJ, Brazil; [2]Laboratório de Oncobiologia Molecular, Departamento de Biotecnologia Farmacêutica, Faculdade de Farmácia, Universidade Federal do Rio de Janeiro, 21941-902, Rio de Janeiro, RJ, Brazil; [3]Instituto de Bioquímica Médica Leopoldo de Meis, Universidade Federal do Rio de Janeiro, 21941-902, Rio de Janeiro, RJ, Brazil; [4]Department of Anatomy, Cell Biology and Physiological Sciences, Faculty of Medicine, American University of Beirut, Beirut, Lebanon.

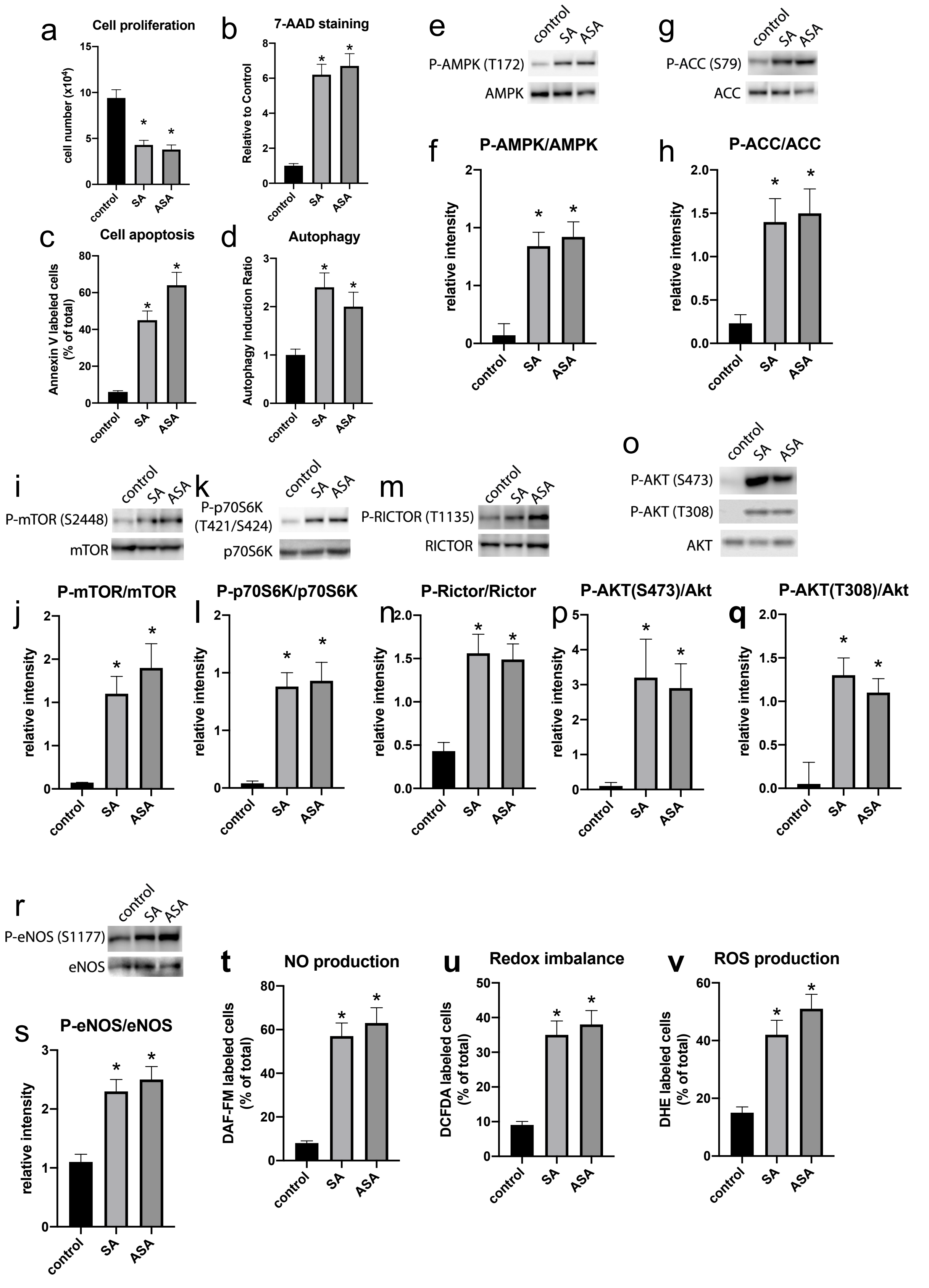

Fig. S1. Cell signaling pathways profiles in B16F10 cells treated or not with SA or ASA. Western blots shown are representative samples of each group that were relatively quantified and represented in the graphics as mean ± S.E.M. of 4 different experiments (n = 4). Panel a: cell

proliferation. Panel b: cell permeation. Panel c: apoptosis. Panel d: autophagy. Panels e and f: AMPK and phospho-AMPK (T172). Panels g and h: ACC and phospho-ACC (S79). Panels i and j: mTOR and phospho-mTOR (S2448). Panels k and l: p70S6K and phospho-p70S6K (T421/S424). Panels m and n: Rictor and phospho-Rictor (T1135). Panels o, p, and q: Akt and phospho-Akt (T308 or S473). Panels r and s: eNOS and phospho-eNOS (S1177). Panel t: NO production. Panel u: oxidative stress evaluation. Panel v: ROS production. * means $P < 0.05$ as compared to the control (One-way ANOVA followed by Dunnett post-test).

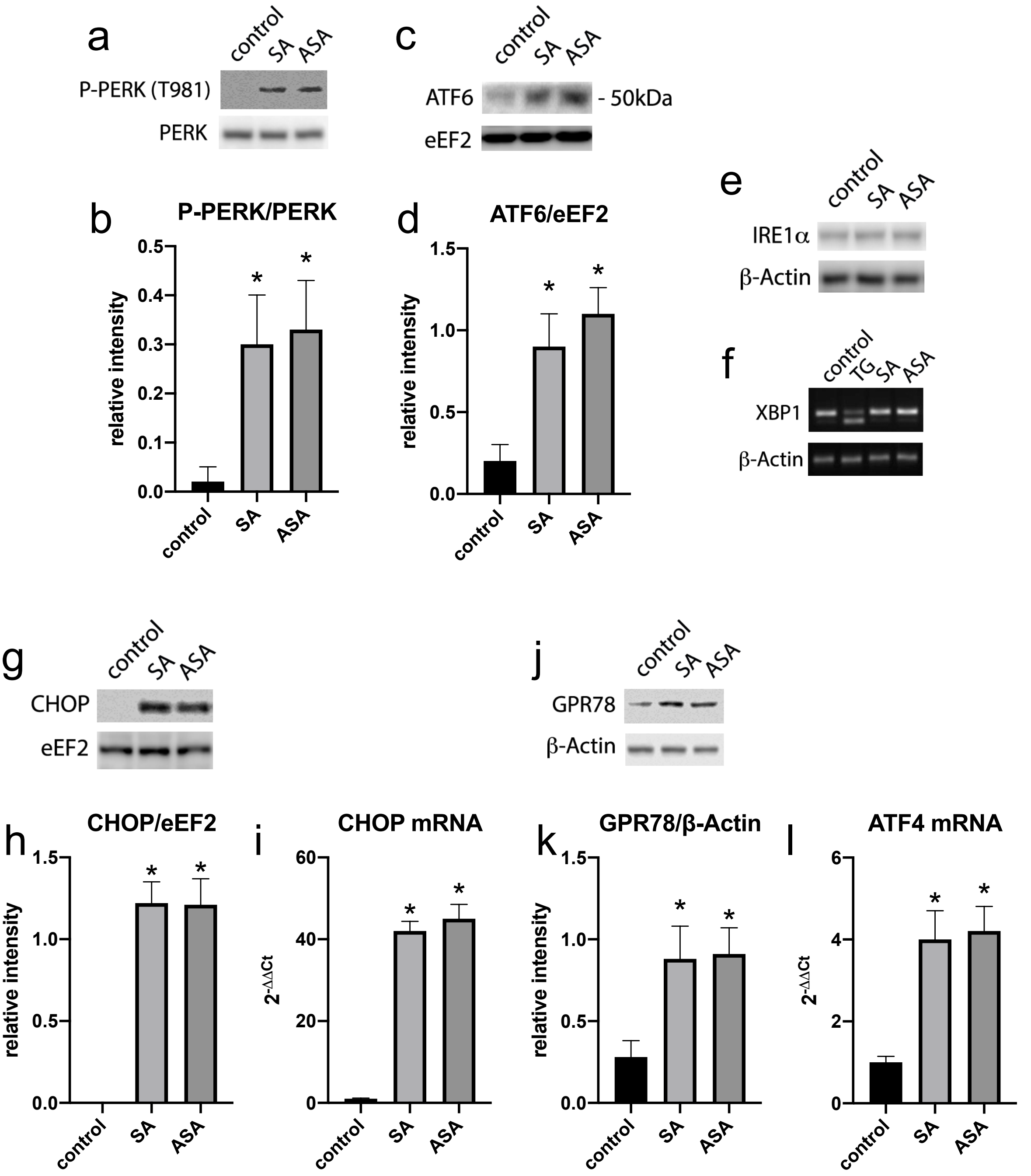

Fig. S2. ER stress response evaluation in B16F10 cells treated or not with SA or ASA. Western blots displayed are the whole processed strips, as indicated in Material and Methods and, such as the PCR, are representative samples of each group and represented in the graphics as mean ± S.E.M. of 4 different experiments (n = 4). qPCR represented in the graphics signify mean ± S.E.M. of 4 different experiments (n = 4). Panels a and b: PERK and phospho-PERK (T981). Panels c and d: ATF6 and eEF2. Panel e: IRE1α. Panel f: XBP1. Panels g and h: CHOP and eEF2. Panel i: qPCR CHOP. Panels j and k: GPR78 and ß-Actin. Panel l: qPCR ATF4. * means $P < 0.05$ as compared to the control (One-way ANOVA followed by Dunnett post-test).

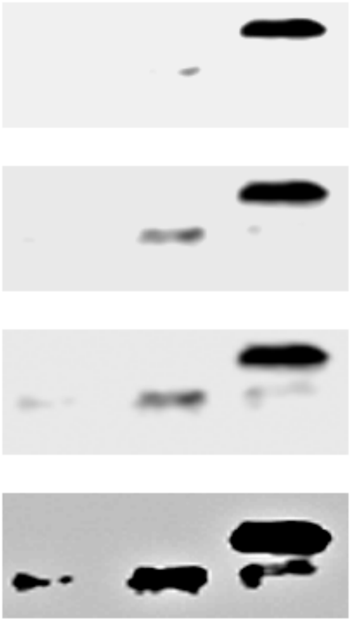

Fig. S3. Different expositions of membrane strip labeling for cleaved caspase 3. Exposition times were 30s, 1 min, 2 min and 5 min, respectively. The 5 min exposition is shown in Fig. 3n. The experiment was performed such as described in the main text of the paper.
